# Murine Typhus in Humans, Yucatan, Mexico

**DOI:** 10.3201/eid1906.121400

**Published:** 2013-06

**Authors:** Karla Dzul-Rosado, Pedro González-Martínez, Gaspar Peniche-Lara, Jorge Zavala-Velázquez, Jorge Zavala-Castro

**Affiliations:** Universidad Autónoma de Yucatán, Yucatan, México

**Keywords:** Rickettsia typhi, human case, flea-borne rickettsioses, murine typhus, Mexico, vector-borne infections, zoonoses

**To the Editor:** *Rickettsia typhi* is the causal agent of murine typhus, a febrile illness affecting humans worldwide (*1*). In Mexico, recent studies demonstrated a 14% prevalence of antibodies against typhus group rickettsiae in healthy adult blood donors in Mexico City, and a recent nonfatal case of endemic typhus was reported in Yucatan (*2*,*3*).

In May 2011, a 42-year-old woman and her 12-year-old son sought care at the clinical service of the Autonomous University of Yucatan. They had malaise, headache, fever (39°C), fibromyalgia, sore throat, and fatigue and an erythematous rash on the chest that after 6 days spread to the abdomen and extremities.

Dengue fever was diagnosed, and the patients were treated empirically with acyclovir, methanesulfonamide, N-(4-nitro-2-phenoxyphenyl) and clarithromycin. Dengue could not be confirmed by laboratory testing.

Murine typhus was diagnosed on the basis of PCR amplification and immunofluorescent assay for antibodies to *R. typhi*. *Rickettsia* species was determined by sequencing of rickettsial genes. Three serum samples were collected from the woman (8, 12, and 16 days after illness onset) and 1 from the boy (8 days) in 3.8% sodium citrate as anticoagulant, and DNA was extracted immediately by QIAamp DNA Blood Mini Kit (QIAGEN Valencia, CA, USA) in accordance with the manufacturer’s instructions. Single-step PCR amplification was performed by using genus-specific primers for the rickettsial 17-kDa protein and citrate synthase (*gltA*) genes as reported (*4*)

Sequences of the citrate synthase and 17-kDa PCR products were compared at the National Center for Biotechnology Information BLAST software (*5*). Three PCR amplicons of both genes were fully sequenced and compared with sequences in GenBank. The 17-kDa and citrate synthase fragment sequences (GenBank accession nos. JX198507 and JX458814) showed 99% and 100% identity, respectively, with *R. typhi* strain Wilmington strain (GenBank accession no. AE017197.1) (Table).

**Table:** Ig detection by immunofluorescent assay and molecular results by PCR and sequence identity of the amplicons of *gltA* and 17-kDa genes of *Rickettsia* spp. from 2 patients with murine typhus, Yucatan, Mexico

Patient age, y/sex	Days after illness onset	Immunofluorescence assay								*Rickettsia* species*
		*R. typhi*			*R. rickettsii*			PCR/sequence test		
		IgM	IgG		IgM	IgG		17 kDa	*gltA*	
45/F	8	256	128		Neg	128		Pos	Pos	*R. typhi*
	12	128	128		Neg	64		Pos	Pos	*R. typhi*
	16	64	128		Neg	64		Neg	Neg	ND
12/M	8	128	128		Neg	64		Pos	Pos	*R. typhi*

*Identified species showed 99% identity with *R. typhi* Wilmington strain 17-kDa antigen gene (GenBank accession no. AE017197.1) and 100% identity with *R. typhi* Wilmington strain *gltA* gene (GenBank accession no. AE017197.1). ND, species not determined.

Immunofluorescent assay was performed by using *R. rickettsii* and *R. typhi* antigen fixed on slides. We examined the serum samples for IgG and IgM, assessing reactivity of γ chain–specific and μ heavy chain–specific secondary conjugates, respectively, with rickettsial antigens. All 3 samples from the woman and the sample from the boy contained antibodies to *R. typhi* (Table). Both patients were treated with 100 mg of oral doxycycline 2×/day for 7 days (boy), and 10 days (woman); symptoms improved in 72 hours for the child. The woman’s symptoms resolved completely in 5 days.

Typhus has been endemic in Mexico since before the conquest period (*6*). Socioeconomic aspects play a major role in zoonotic diseases, such as rickettsioses, especially in their distribution in urban and suburban areas because of factors such as marginalized communities, animal breeding, education levels, poverty, and social exclusion from health systems.

Overcrowding resulting from migration from rural areas to large urban centers contributes to increased zoonoses in urban areas. Also contributing is the ecologic imbalance of flora and fauna associated with deteriorating sanitary conditions in areas where mammals involved in the cycle of *R. typhi*, such as rodents and opossum, may live in the same habitat as humans and colonize backyards, waste deposit area, and areas around the neighborhoods where they can find food. The concurrence and presence of mammals, vectors, and humans may contribute to maintaining transmission of endemic typhus in a reduced area, with the possibility to cause outbreaks (*7*,*8*). Housing conditions and culture, such as courtyards with vegetation and presence of pets, in several suburban areas of Yucatán encourage close contact between humans and possible reservoirs for several infectious diseases, such as mice, rats, opossums, dogs and cats, and their ectoparasites (*9*).

Because housing and cultural conditions are similar in all countries of Latin America, endemic typhus probably is transmitted in the same way: by coexistence with domestic animals and close contact with wild reservoirs. Epidemiologic control should be closely linked to education aimed at encouraging villagers to interrupt the cycle of transmission and thus prevent the disease.

We have confirmed the presence of murine typhus in Yucatan. The finding of human cases demonstrates the need to consider *R. typhi* infection in the differential diagnosis of febrile illnesses considered endemic in Yucatan, such as dengue fever and leptospirosis (*10*). Early and accurate diagnosis should enable physicians to treat this disease appropriately and early in the clinical course to prevent increased illness and death.
